# Is back pain during childhood or adolescence associated with muscle strength, muscle endurance or aerobic capacity: three systematic literature reviews with one meta-analysis

**DOI:** 10.1186/s12998-015-0065-8

**Published:** 2015-07-16

**Authors:** Arnaud Lardon, Charlotte Leboeuf-Yde, Christine Le Scanff

**Affiliations:** EA 4532 CIAMS, Université Paris-Sud, UFR STAPS, 91405 Orsay, France; Institut Franco-Européen de Chiropraxie, 24 Bld Paul Vaillant Couturier, 94200 Ivry sur Seine, France; Research Department Spine centre of Southern Denmark Hospital, Hospital Lillebælt Middelfart, Institute of Regional Health Research, University of Southern Denmark, Odense, Denmark

**Keywords:** Back pain, Adolescent, Children, Back muscle endurance, Back muscle strength, Aerobic capacity, Meta-analysis, Systematic review

## Abstract

**Background:**

Back pain is a common condition during childhood and adolescence. The causes of back pain are largely unknown but it seems plausible that some physical factors such as back muscle strength, back muscle endurance and aerobic capacity may play a role in its development, in particular in the early years.

**Objectives:**

The objectives of this review were to investigate in childhood and adolescence 1) if muscular strength in trunk extension is associated with back pain, 2) if muscular endurance in trunk extension is associated with back pain and 3) if aerobic capacity is associated with back pain.

**Design:**

Three systematic critical literature reviews with one meta-analysis.

**Methods:**

Systematic searches were made in June 2014 in PubMed, Embase and SportDiscus including longitudinal, retrospective or cross-sectional studies on back pain for subjects <20 years. Articles were accepted if they were written in French or English. The review process followed the AMSTAR recommendations. The possibility of conducting a meta-analysis was assessed for each research question.

**Results:**

Four articles were included for the first objective, four for the second and three for the last. None of the included articles found an association between back muscle strength in extension and back pain. For the second objective, a protective association between back muscle endurance in extension and back pain was found, later confirmed in a meta-analysis (OR = 0.75, 95 % CI 0.58-0.98). The association between aerobic capacity and back pain is not clear.

**Conclusions:**

High back muscle endurance in extension appears protective of back pain in youngsters, but the roles of high back muscle strength in extension and aerobic capacity are less clear.

## Background

Pain is relatively common in childhood and adolescence [1]. For example, in a population of circa 3000 adolescents, 61 % reported musculoskeletal pain at least in one area [2]. Back pain (BP) was noted to be the second most common type with 25 % reporting daily complaints [2]. BP is common during childhood and has been shown to be a predictor of low back pain (LBP) in adulthood [3]. Therefore, more knowledge is needed about BP in the early years, as attention needs to be focused on this period of life.

It is well known that physical activity has a positive effect on health [4] by decreasing adiposity and improving cardiovascular health, cardiovascular fitness, mental health, academic performance, musculoskeletal health, fitness and bone mineral density [5]. It seems likely that physical activity, through its positive influence on back muscle strength (BMS), back muscle endurance (BME) and aerobic capacity (AC) could also improve spinal health. However, physical activity is not the only factor which could have an effect on BMS, BME and AC during childhood and adolescence. The body composition changes during this growing period [6, 7], which could also have an effect on BMS [8] and AC [9]. There is conflicting evidence on the importance of these factors in adulthood [10].

BMS is the force generated by contraction of back muscles, whereas BME is the capacity of these muscles to sustain a sub-maximal contraction force for as long as possible (through isometric contraction). AC, on the other hand, reflects the capacity of cardio-vascular endurance [11].

BMS is usually measured in Newtons using a dynamometer while the participant performs isometric, isotonic or isokinetic maximum voluntary contraction [12]. For lumbar spine muscles, the duration of isometric contraction in extension, i.e. BME, is often measured with the Biering-Sorensen test, which has been shown to have good reliability (ICC = 0.77; 95 % CI, 0.52-0.90) [13]. AC can be measured through various cardio-vascular endurance tests, such as running or biking, by estimation of the VO_2max_, which is the maximum volume of oxygen consumed in one minute at maximum effort. It is also possible to calculate the VO_2max_ from other tests, such as 20 m shuttle run that measures the performance [14].

Only one systematic review appears to have been published on physical fitness and LBP in youth [15], but it did not specifically address BMS, BME, and AC. It is not clear if physical training, which would result in better strength and endurance, could have a preventive effect on BP in young people.

In order to obtain an overview of the present status of the scientific literature on the topic of muscle strength and endurance in relation to BP in youngsters, three systematic literature reviews were performed. The specific research questions addressed were:

1. Is muscular strength in trunk extension associated with BP?
2. Is muscular endurance in trunk extension associated with BP?
3. Is aerobic capacity associated with BP?

## Method

### Design

Three systematic critical reviews were carried out following the criteria listed in AMSTAR [16]. In addition, a meta-analysis was performed for the second research question. The review was registered on PROSPERO international prospective register of systematic reviews (PROSPERO 2014: CRD42014006189).

### Search

Searches were performed in Pubmed, Embase and SportDiscus databases in June 2014 without time limit. The following search terms were used as free text or MeSH terms: muscle strength, muscle endurance, isokinetic, isometric, power, maximum voluntary contraction, muscle fatig*, aerobic capacity, aerobic fitness, maximum oxygen consumption, cardiovascular fitness, endurance, physical fitness, back pain, backache, spinal pain, children, adolescent, teen. The search strategy was designed in collaboration with a librarian from the University of Paris-Sud.

### Eligibility criteria

The eligible studies included in this review were longitudinal, retrospective or cross-sectional. We selected only articles written in English or French. The study population of selected studies had to be below the age of 20 (to include mainly participants who were not yet fully grown) and the sample size had to be superior to 100 at baseline. The target condition, BP, should not be included in a generic term only, such as a musculoskeletal pain. Studies relating only to the neck were not eligible. We excluded case reports, studies where only muscles other than back muscles were included and studies in which the BMS, BME and AC were not objectively measured. We required that the BMS was measured with a dynamometer, the BME assessed with the Biering-Sorensen test or by another test with the same reliability and that the AC was measured either in laboratory to obtain the VO_2max_, or by the two following field tests: PW170 or 20 m shuttle tests; both shown to be valid predictors of VO_2max_ in adolescents [17].

### Screening

**Fig. 1:**
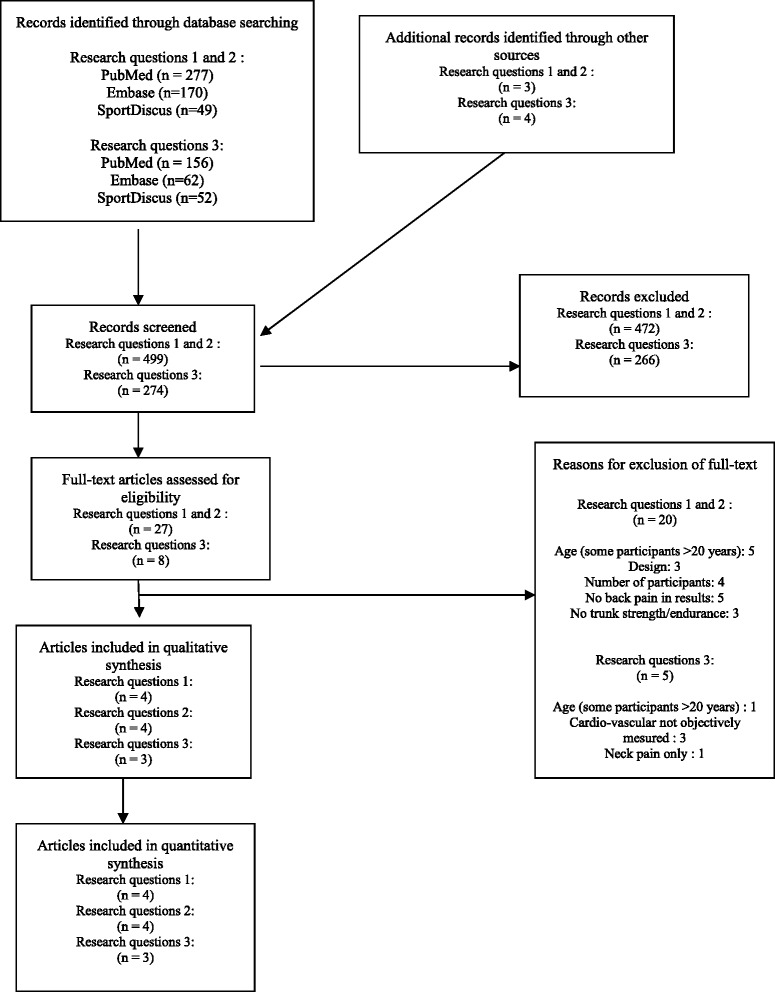
PRISMA 2009 flow diagram

The first author made the search in the databases and selected the potentially relevant full texts from titles and abstracts. This selection was repeated three months later to be sure that the first author could not remember the first selection (i.e. blind to his first choices), with the same results. The same investigator screened if other references could be found by tracking references from articles. The first and second author independently assessed if articles could be included in the review according to the inclusion and exclusion criteria based on the full texts. The selection process is summarised in Fig. 1, according to the PRISMA 2009 flow diagram [18]. The reasons for exclusion are specified in this diagram. The selected articles were then divided between the three topics.

### Methodological quality assessment

As shown below, a checklist from a previous review was used [19], derived from the checklist of Landrivon [20]. The first and second authors completed the checklist independently for all included articles and compared their results. The quality assessment items selected for this review process were relevant for the purposes of the present study, but would not necessarily reflect the quality in relation to the authors’ original research question(s).

The checklist (annexe 1) contained six main topics (the first four related to the method of the studies and the two last concerned the results and whether these were subjected to relevant multivariate analyses):

1. Study sample. The representativeness of the sample in relation to the corresponding target population was assessed to know if it would be possible to generalize the results to the general population. In cases where the response rate was inferior to 80 %, we assessed if authors investigated potential response bias.
2. Data collection. In order to limit expectation bias, data on back pain and BMS/BME/AC should be collected by two different persons, blind to each other’s findings, unless at least one of these items was collected in a questionnaire.
3. The studied factor: BMS/BME/AC. This factor should be clearly defined, i.e. explaining how the data was obtained and using a measure, stated to be valid and/or reliable.
4. The outcome measure: back pain. The outcome measure should be clearly defined. The way back pain was assessed should be clearly explained and the recall period should be less than 1 month to limit memory decay [21].
5. Results of the study. The presence or absence of association was recorded.
6. Multivariate analysis. It was explored if the results remained the same after controlling for other relevant variables (i.e. age, sex)

### Data analysis and synthesis

The three reviews were performed separately but using the same procedure, as described above. The results of the methodological assessment were summarised in Tables 1, 2, 3; one table for each research question. A methodological quality score was obtained for each article. No cut-point for level of quality was established.

**Table 1:** Methodological checklist objective 1

Ref numb.	Name of first author/year	Study sample	Data collection	Studied factors: TMS	Back pain	Score and %
[24]	Balagué 1993	N-N	Y	Y-Y-Y	Y-Y-Y	7/9 78 %
[25]	Balagué 2010	N-N	?	Y-Y-Y	Y-Y-N	5/9 56 %
[26]	Merati 2004	Y-N	Y	Y-Y-Y	Y-Y-N	7/9 78 %
[27]	Newcomer 1996	N-N	?	Y-Y-Y	Y-Y-N	5/9 56 %

**Table 2:** Methodological checklist objective 2

Ref numb.	Name of first author/year	Study sample	Data collection	Studied factors: TME	Back pain	Score and %
[28]	Andersen 2006	Y-Y	Y	Y-N-N	Y-Y-Y	7/ 78 %
[29]	Bernard 2007	Y-N	Y	Y-N-N	Y-Y-NA	5/8 63 %
[30]	Perry 2009	Y-N	Y	Y-Y-Y	Y-Y-Y	8/9 89 %
[31]	Johnson 2009	Y-N	Y	Y-Y-N	Y-Y-Y	7/9 78 %

**Table 3:** Methodological checklist objective 3

Ref numb.	Name of first author/year	Study sample	Data collection	Studied factors: AC	Back pain	Score and %
[28]	Andersen 2006	N-Y	Y	Y-N-N	Y-Y-Y	6/9 67 %
[32]	Cardon 2009	Y-N	Y	Y-Y-Y	Y-Y-Y	8/9 89 %
[30]	Perry 2009	Y-N	Y	Y-Y-Y	Y-Y-Y	8/9 89 %

The results were summarised in Tables 4, 5, 6. All the BP variables listed in the method section of the included articles were included in these tables, even if they were not reported in relation to the independent variables (BME, BMS, and AC).

**Table 4:** The association between back muscle strength in extension and back pain in people younger than 19 years as reported in four studies

Ref numb.	Name of first author/year	Design of study	Independent variable	Dependent variables listed in method	Dependent variables reported in result section	Association BMS in extension and back pain
[24]	Balagué 1993	CS	BMS in N	Cumulative life prevalence	History of BP	None
				Point prevalence		
				Localization of BP		
[25]	Balagué 2010	CS and P	BMS in N	LBP medical attention	History of LBP	None
				LBP consequences		
				Last episode		
				Parental history of BP		
[26]	Merati 2004	CS	BMS in N	Frequency of BP	History of BP	None
[27]	Newcomer 1996	P	BMS in N	LBP-ever	LBP-ever	None
				Age of first episode	Age of first episode	
				One-Year prevalence	One-Year prevalence	
				LBP consequences	LBP consequences	
				LBP-doctor	LBP-doctor	

CS: cross-sectionnal study

P: prospective study

BMS: back muscle strength (in extension)

N: Newton

BP: Back pain

LBP: Low back pain

**Table 5:** The association between back muscle endurance in extension and back pain in people younger than 19 years as reported in four studies

Ref numb.	Name of first author/aawyear	Design of study	Independent variable	Dependent variables listed in method	Dependent variables reported in result section	Association Trunk Muscle Endurance in extension and BP		
[28]	Andersen 2006	CS	Time, obtained by Sorensen-Biering’s test	LBP/MBP/NP < 1 month Age at 1^st^ BP Consequences	BP last month	YES, isometric endurance in the trunk muscle was negatively associated with back pain after adjusting for height and sex		
						Odds ratio: Three upper quartiles Vs lowest quartile		
						OR = 0.89 (95 % CI, 0.78-1.02)		
						OR = 0.78 (95 % CI, 0.68-0.89)		
						OR = 0.71 (95 % CI, 0.62-0.82)		
[29]	Bernard	RS	Time, obtained by Sorensen-Biering’s test	CLBP Subgroup within control group with LBP	CLBP	YES, the endurance in back extensors were less in the CLBP group		
						Sorensen Median(min:max) (25 s:3 min43)	Control group 2 min31 (32 s:10 min2)	CLBP 1 min45
[30]	Perry 2009	CS	Time, obtained by Sorensen-Biering’s test	BP pain (without NP) Ever Month Chronic Diagnosed	BP pain Ever Month Chronic Diagnosed	NO for male Yes, for girls. Increased likehood of diagnosed back pain was associated with reduced back endurance (OR = 2.05; 95 % CI, 1.16-3.60) and greater back endurance (OR = 2.00; 95 % CI, 1.10-3.60)		
[31]	Johnson 2009	CS	Time, obtained by Sorensen-Biering’s test of static muscular endurance	1-year BP Present BP	1-year BP Present BP	YES Patient without LBP had a significant higher isometric holding time than those with history of previous LBP and those with present LBP.		

CS: cross-sectionnal study

RS: retroprospective study

BMS: back muscle strength (in extension)

BP: Back pain

LBP: Low back pain

CLBP: Chronic low back pain

**Table 6:** The association between aerobic capacity and back pain in people younger than 19 years as reported in four studies

Ref numb.	Name of first author/year	Design of study	Ind. VAR	Dependent VAR possible to use	D VAR used in result section	Association aerobic capacity and BP			
						Male		Female	
[28]	Andersen 2006	CS	V0_2_ max ergometer	LBP/MBP/NP < 1 month Age of 1^st^ BP Consequences	BP last month	Both sexes:			
						YES, in bivariate analysis: best quartile Vs least quartile			
						OR = 0.81			
						This association disappeared when adjusted for back trunk muscle endurance			
[32]	Cardon 2009	CS	Endurance shuttle run	LBP/DP/NP Last week Severity Frequency	Pain/no pain	Yes		No	
						F (p) 4.1 (0.04)		F (p) 0.3 (0.59)	
[30]	Perry 2009	CS	Cycle ergometer	BP without NP Ever Month Chronic Diagnosed	BP without NP Ever Month Chronic Diagnosed	B	M	B	M
						Yes	No	No	No
						Yes	Yes*	No	No
						No	No	No	No
						No	No	No	No
						HQR vs IQR: OR = 1.53 (95 % CI 1.08-2.17)*			

CS: cross-sectionnal study

BMS: back muscle strength (in extension)

BP: Back pain

LBP: Low back pain

DP: Dorsal pain

NP: Neck pain

B: Bivariate analysis

M: Multivariate analysis

HQR: 75 % high quartile

IQR: Interquartile

F(p): difference between group (*p*-value)

According to the type of results reported in the articles, the possibility of conducting a meta-analysis was assessed for each research question. Meta-analysis could be performed only if appropriate information was available in each included study. To perform the meta-analysis, we used a random effect model because the samples of the included studies did not emanate from the same underlying study population. Only one outcome variable can be used in a meta-analysis because the same participants cannot be included in the analyses more than once. It is also necessary to select only one outcome variable because the same participants cannot be included in the analyses more than once. Therefore, if several definitions of BP were available in a study, whenever possible, the one-month prevalence estimate was selected for the subsequent analyses.

Our hypotheses were that a high BMS, BME or AC has a negative association with BP. In the statistical model, we compared the middle or lower values against the highest 25 % quartile. If results were presented only as means with standard deviations (SD), the standardised mean differences (SMD) and their respective SD were calculated enabling the estimation of odds ratios OR (lnOR = (π/√3) x SMD) [22]. The heterogeneity across the studies was described as the I^2^ [23]. We did not implement a cut-point for heterogeneity. Instead, if a large heterogeneity was found, we attempted to find explanations for this based on the method of the studies.

## Results

### Number of articles

For the first two research questions, 496 records were identified: 277 in PubMed, 170 in Embase and 49 in SportDiscus. Of these, 40 full texts were selected, 13 of which were duplicate studies resulting in 27 relevant full texts that were assessed for eligibility. Ultimately eight articles were included in the review, four of them relating to question 1 [24–27] and four to question 2 [28–31]. A hand search resulted in the identification of three potential texts, none of which were suitable for inclusion.

For the third research question, 270 records were identified: 156 in PubMed, 62 in Embase and 52 in SportDiscus. Eleven relevant full texts were selected, three of which were duplicate studies, resulting in eight full texts that were assessed for eligibility. Three of these were suitable for inclusion [28, 30, 32]. A hand search resulted in the identification of four potential texts, none of which were included. The reasons why these articles were excluded are listed in Fig. 1.

Two articles [28, 30] included information pertinent to two of the three objectives. In the tables and results section, articles were listed in alphabetic order.

### A. Is muscular strength in trunk extension associated with BP?

#### Description of studies or BMS

Four articles were included to answer the question of whether BMS in trunk extension is associated with BP. The first article, Balagué *et al.* [24], presents a cross-sectional study in which 117 children aged 11 to 15 years (response rate: 97 %) were included. Its purpose was “to evaluate the relationship between the dynamic strength profile of the trunk, anthropometric parameters, BP and frequency of sport activities performed”. BMS was evaluated using an isokinetic Cybex II dynamometer. Information on BP was obtained through interview and defined in relation to location, cumulative life prevalence and point prevalence. The association between BMS and presence of past history of BP was studied controlling for self-reported frequency of sport. Univariate, multivariate and correlation analyses were used to determine the association between BMS and BP.

The second article, Balagué *et al.* [25], reports the results from a cross-sectional and a prospective study, in which 95 children aged 13 to 14 years remained at follow-up (response rate: not reported). The objective was to examine if trunk performance capacity has an association with LBP in adolescent boys. The trunk muscle performance was evaluated with standard dynamometer testing protocols. The presence of LBP was determined using a brief semi-structured interview with questions about medical attention and the time of the last episode. The difference in BMS was studied for the groups with and without LBP. The unpaired t-test was used to compare BMS in those with and without BP.

The third article, Merati *et al.* [26], presents a cross-sectional study in which 144 12-year olds were included (response rate: not reported). The goal of this study was to assess if a deficit in trunk muscular strength plays a role in BP occurrence in pre-pubertal subjects. BMS was measured with a modular-components isokinetic dynamometer. A questionnaire was used to determine the presence of BP. The student t-test was used to compare BMS in those with and without BP.

The fourth article, Newcomer and Sinaki [27], presents a prospective study with a four-year follow-up in which 96 study subjects, aged from 10 to 19 years, remained in the final study group (response rate: 39 %). The main purpose was to determine the occurrence of LBP and its relationship to back strength and physical activity in children. Back strength was tested at baseline by an iso-dynamometer. LBP at follow-up was determined in an interview, based on a list of five questions about LBP-ever, the age at the first episode, one-year prevalence, the consequences on school and sport activities and medical attention. To evaluate this association, logistic regression was used.

#### Quality assessment of articles on BMS

The quality scores in the four reviewed articles were 56 %, 56 %, 78 % and 78 % (Table 1).

##### Studied factor: back muscle strength

All the articles assessed BMS with a dynamometer. In one study [25], reference was made to a previous study having shown the measurement to be reliable whereas in two of the studies, reliability was tested and shown to be acceptable in one [24], but results were unreported in the other [26]. In the fourth study [27], the dynamometer was calibrated and it was reported that the method had been previously shown to be reliable and valid. In general, these data can therefore probably be trusted.

##### Outcome measure: back pain

BP was clearly defined in all the articles as well as the description of the BP assessment. However, only one article reported a recall period of one month or less [24], which was considered suitable in young people. The other articles reported in their results section a history of LBP [25], a recall period of six months [26], and recall periods of one year and a lifetime [27].

##### Data collection

In two articles [24, 25], the data collection for BP was made through semi-structured interview but it was not clear if the person who made the interview and the person in charge of strength measurement were the same. In the other two [26, 27], questionnaires were used, thus ensuring separate data collection of these two variables, necessary to prevent reporting bias.

##### Study sample

All studies recruited at least some of their study subjects from schools, one having to resort to additional assistance from medical practitioners for recruitment [24]. In only one of the studies [26],participants were reported to have been randomly selected. Whether study participants were representative of the general population is therefore doubtful.

#### Results for research question 1

None of the four relevant articles demonstrated an association between BMS in extension and BP. Therefore no meta-analysis was performed for this research question.

### B. Is muscular endurance in trunk extension associated with BP?

#### Description of studies on BME

The first article exploring the association between BME in trunk extension and LBP reports on a cross-sectional study written by Andersen *et al.* [28], in which 9413 17-year olds were included (response rate: 41 %). The aim was “to examine the association between physical fitness and self-reported BP in adolescents”. The BME was assessed with the Biering-Sorensen test. BP was self-reported and focused on the presence of pain in the past month, prior experience of BP and the location of the pain. Logistic regression was used to assess the association between BME and BP adjusting for sex, height and smoking.

The second article, Bernard *et al.* [29]*,* describes a retrospective study in which 327 individuals aged 10 to 18 were included (response rate: 50 %). The main aim was “to compare muscle endurance of back flexors and extensors between a control group of 276 teenagers and a group of 51 teenagers from a pediatric unit, who suffered from chronic LBP”. The BME was assessed with the Biering-Sorensen test. LBP information was assessed with a visual analogue scale in a specific questionnaire for the chronic LBP group. The relevant analysis was performed by comparing the BME in the clinical group to the control group. Nevertheless, some of the participants in the control group also reported some LBP (n = 47 according to the method section and n = 48 according to the results section). How this information was obtained was not explained. The association between BME and BP was tested using linear regression.

The third article, Johnson *et al.* [30], is a cross-sectional study including 625 youngsters aged 11-19 (response rate: not reported). The aim was “to establish reference data and pattern of back extensor strength in school-aged Nigerian adolescents”. The BME was assessed with the Biering-Sorensen test. The history of LBP and present LBP was assessed by questionnaire. The difference in BME was tested for those with or without a history of LBP using a t-test. The same was done for present LBP.

The fourth relevant article, Perry *et al.* [31], also describes a cross-sectional study in which 1608 adolescents, all aged 14, were included (response rate: 69 %). The aim of this study was to determine if physical fitness is related to increased risk of BP. The BME was assessed with the Biering-Sorensen test. Information on BP was obtained by a questionnaire including lifetime prevalence of pain, pain in the last month, chronic pain and also pain diagnosis. Results were reported separately for boys and girls, in which the lower 25 % and the higher 25 % were compared to the middle 50 %. The association between BME and BP was tested with multivariate logistic regression.

#### Quality assessment of articles on BME

The methodological quality scores were 67 %, 78 %, 78 %, and 89 % (Table 2).

##### Studied factor: back muscle endurance

All the authors assessed BME with the Biering-Sorensen test, which has been reported to be a reliable and valid tool [13, 33].

##### Outcome measure: back pain

The definition of BP and method of assessment were always clearly defined. In all articles except one [29], the recall period was appropriate for at least one variable concerning BP. However, the aim of that article was in fact to compare a clinically affected group of children with chronic LBP against a group of “normal” children. The recall period, therefore, did not appear to be of importance in this case.

##### Data collection

The data on BME and BP were collected independently (blindly) by two different persons or at least by using a questionnaire in all studies.

##### Study sample

Although attempts were made to access children from the general population, in three of the studies representativeness was not explicitly addressed [29–31]. In the fourth study [28], although the target population was not representative of the general population, their sample was compared to another representative group and no difference was found in the physical fitness test between these two groups, meaning that their study sample had external validity, at least on this key variable.

#### Results for research question 2

In all four articles, an association was found between BME and BP. In three of these [28–30], it was reported that those with BP had a weaker BME compared to those without BP (Table 5). In the fourth study [30], many associations were tested. In this study, only one (diagnosed BP) of four outcome variables (BP ever, one month prevalence, chronic back pain (CBP), diagnosed BP) was statistically significant for girls and not for boys. In the text, multivariate analysis is reported to have resulted in an increased likelihood of diagnosed BP in those with reduced BME as compared to the middle group. On the other hand, those with the greater BME, when compared to the middle group, were also found to be more likely to report diagnosed BP, i.e. indicating a U-curve for diagnosed BP.

**Fig. 2:**
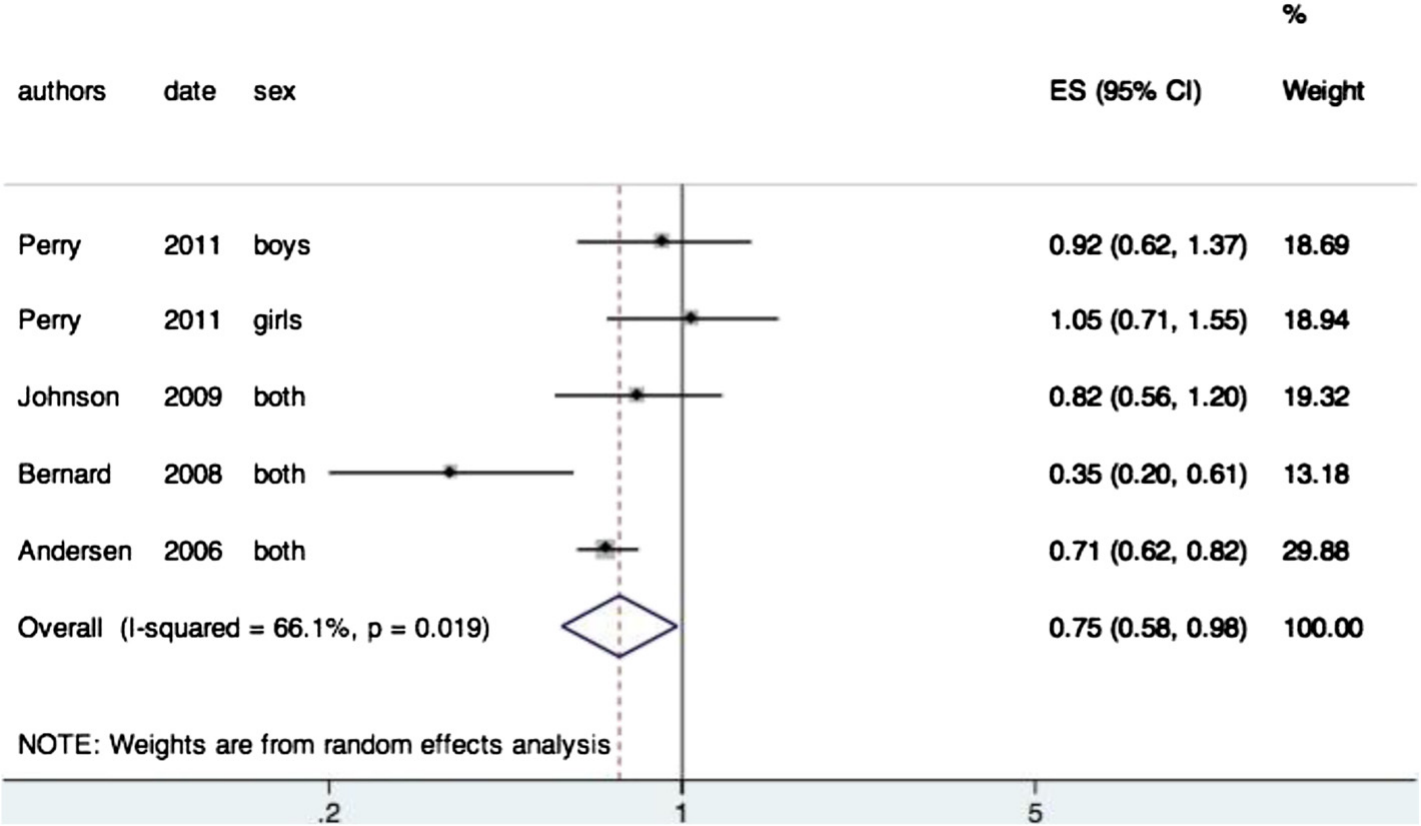
Is muscular endurance in trunk extension associated with BP? Meta-analysis both = girls and boys

On this topic, in all articles, the data allowed us to perform a meta-analysis (Fig. 2). A negative association was found between the BME and BP (OR = 0.75, 95 % CI 0.58-0.98). The I^2^ was 66.1 % indicating a high heterogeneity between the studies. This can be explained by the fact that some articles divided their sample according the sex of the participant and by the differences in the definition of back pain.

### C. Is aerobic capacity associated with BP?

#### Description of studies of AC

The first of the three relevant articles for the third research question, relating to AC in general and BP, reports the results from a cross-sectional study performed by Andersen *et al*. [28], in which 9413 17-year olds were included (response rate: 41 %). The aim was “to examine the association between physical fitness and self reported BP in adolescents”. AC was assessed by VO_2max_ measured with the help of a cycle ergometer. BP was self-reported and defined as the presence of pain in the last month, prior experience of BP and the location of the pain. Logistic regression was used to assess the association between BME and BP adjusting for sex, height and smoking.

The second article, Cardon *et al.* [32], describes a cross-sectional study in which 749 children aged 8 to 12 years were included (response rate: not reported). The aim of this study was “to examine whether physical fitness, physical activity, and psychosocial determinants of physical activity are associated with reports of back or neck pain”. AC was assessed by a 20-m endurance shuttle run protocol. BP was evaluated with a questionnaire that defined BP based on the location of pain in the past week. It also included severity and frequency. Analysis of variance was used with age as a covariate to determine the association between AC and BP.

The third article for this research question, Perry *et al.* [30], also describes a cross-sectional study in which 1608 14-year olds were included (response rate: 69 %). The aim of this study was to determine if physical fitness is related to increased risk of BP. The AC was assessed by sub-maximal cycle ergometry, using a specific protocol (PWC 170). Information on BP was obtained with a questionnaire that included lifetime prevalence of pain, pain in the last month, chronic pain and also pain diagnosis. Results were reported separately for boys and girls, in which the lower 25 % and the higher 25 % were compared to the middle 50 %. The association between AC and BP was tested with multivariate logistic regression.

#### Quality assessment of articles on AC

The three articles had quality scores of 67 %, 89 %, and 89 % (Table 3).

##### Studied factor: aerobic capacity

All the authors clearly defined the tools used to evaluate AC and all but one [28] provided references about validity and reliability of their evaluation test.

##### Outcome measure: back pain

BP (and the way it was assessed) was clearly defined in all the articles. The recall periods were one month or less in all studies [28, 32], although Perry *et al.* [30] also used longer recall periods.

##### Data collection

In all the studies, the data for BP were collected with a questionnaire and therefore the independent and dependent variables were collected separately.

##### Study sample

Attempts were made to access children from the general population in all three studies. As previously explained, in one of the studies [28] the results on the physical fitness tests were similar to those in a representative sample from another study. For the other two [30, 32] the final representativeness is unknown.

#### Results for research question 3

In all three articles, at least one association was reported between AC and BP. In two of the studies, results were reported separately for girls and boys with positive findings only for the boys [30, 32]. However, these two studies reported conflicting results as one study found a positive association [30] whilst the association was negative in the other [32]. In the third study [28], the association between AC and BP disappeared after adjustment for BME. Hence, the AC and BP may well be associated but it is unclear how, with the possibility that AC is but a proxy for BME. Meta-analysis was not performed for this research question because it was not possible to obtain the confidence intervals of all the odds ratios in the included articles.

## Discussion

### Summary of findings

To our knowledge, this is the first review to explore status of the literature on the associations between back problem and BMS in trunk extension, BME in trunk extension and AC, during childhood and adolescence. No association could be found between BMS in extension and BP. However, the current research suggests that the two other components, BME and possibly AC, have an association with BP. Results were relatively homogeneous between studies and, therefore, we did not interpret the findings in relation to the level of quality or methodological approach between the studies. It is important to note that in one article [30], four variables for BP were tested (ever/month/chronic/diagnosed) and a positive association was found for only one of these variables.

### Methodological considerations of own review

As in all systematic reviews, it is possible that some articles were not captured, either through the search strategy or when selecting the final texts. However, we sought the help of a professional librarian for the search and we did a double-screening of titles and abstracts to limit this risk.

A specific checklist published in a previous study was used for the quality assessment but an emphasis could have been put on other issues, which might have changed our approach to this topic. Also, we studied only extension of the lumbar spine. Other directions of movement or other spinal areas could perhaps resulted in other findings.

### Methodological consequences of reviewed articles

Our systematic reviews were designed to determine an association and not causality. The reason for this was that the cross-sectional design of the included studies does not make it possible to study causality between the physical factors and BP. For this, prospective studies are needed, and further, study subjects should be back pain-free at baseline. It is, therefore, not possible to determine the direction of events (if any) between the physical status and BP. Nevertheless, now that a statistical link has been established between BME and BP, it would be relevant to perform well-designed prospective studies, to investigate which precedes the other.

Another limitation was that none of the reviewed articles took into account the potential modifying effect of growth and physical development.

In the meta-analysis, the score of the I^2^ is high (66 %) and revealed a heterogeneity between the included studies. This heterogeneity could be explained by several factors such as differences in age, recall period and study sample. The validity of the results may hence be limited. On the other hand, if the outcome is apparent despite the differences between studies, this could indicate that the association is indeed solid across populations and definitions of variables.

Aerobic capacity seems to be linked with BP because the three included studies found at least one association. However, in the article [28], in which the results were adjusted on BME, this association disappeared after the adjustment. Unfortunately, the other studies did not adjust for this. It appears reasonable that BME and AC are two expressions of body build, in which case a genetic background may well be of interest.

### A discussion of results in relation to other literature

Our results are in disagreement with a previous systematic review [10] in an adult population based on prospective studies, in which inconclusive evidence for a relationship between BMS and BP and strong evidence was noted that there is no relationship between BME and future LBP. As the time around puberty has been shown to be the period during which BP develops [34], it would be difficult to discover an association between a real risk factor and BP if this link is confused by many other contributing factors later in life. In other words, even if prospective studies are carried out but the baseline population consists of adults, it is probably too late to develop incident BP, which could explain the lack of association in the adults.

On the other hand, if their observations hold true also in youngsters, a credible explanation would be a reversed cause, *i.e*. BP causes decreased BME and AC and not the opposite.

## Conclusion

The present review revealed there to be no association between increased BMS in trunk extension and BP, whereas such an association was clearly present when testing for BME. When adding the results of the meta-analysis for the BME data, the previous findings were confirmed that there is a small but statistically significant protective effect of BME on BP. However, the association between AC and BP requires further studies to evaluate if there is a modifying or confounding link with BME.

**Table:** 

1. Study sample	- Was the sampling likely to be suitable to obtain a group representative of the corresponding general population?
	- If participation at BL or at FU<80 % or unreported, was response bias investigated?
2. Data collec-tion	- Data for BP and independent variable were collected independently by 2 different persons or by at least 1 questionnaire?
3. TMS, TME, AC	- Clearly defined?
	- Reference provided for validity of test?
	- Reference provided for reliability of test?
4. Back pain	- Clearly defined?
	- Description of how BP was assessed?
	- Recall period (<= 1 month)?
5. Stat. analysis	- Was there a positive association between independent and dependent variables ?
6. Multivariate analyses	- If yes, did this association remain after controlling for other relevant
Score	

## Abbreviations

BP: Back pain
LBP: Low back pain
BMS: Back muscle strength
BME: Back muscle endurance
AC: Aerobic capacity
OR: Odds ratio
